# Ultrasonic Monitoring of the Water Content in Concentrated Water–Petroleum Emulsions Using the Slope of the Phase Spectrum

**DOI:** 10.3390/s22197236

**Published:** 2022-09-24

**Authors:** Ediguer E. Franco, Carlos A. B. Reyna, Alberto L. Durán, Flávio Buiochi

**Affiliations:** 1 Engineering Faculty, Universidad Autónoma de Occidente, Cll 85 # 115-85, Cali 760030, Colombia; 2 School of Engineering, University of São Paulo, Avenida Prof. Luciano Gualberto 380, São Paulo 05508-010, Brazil

**Keywords:** ultrasound, backscattering, phase slope, volume fraction, water–petroleum emulsion

## Abstract

This work proposes the slope of the phase spectrum as a signal processing parameter for the ultrasonic monitoring of the water content of water-in-crude oil emulsions. Experimental measurements, with water volume fractions from 0 to 0.48 and test temperatures of 20 °C, 25 °C, and 30 °C, were carried out using ultrasonic measurement devices operating in transmission–reception and backscattering modes. The results show the phase slope depends on the water volume fraction and, to a lesser extent, on the size of the emulsion droplets, leading to a stable behavior over time. Conversely, the behavior of the phase slope as a function of the volume fraction is monotonic with low dispersion. Fitting a power function to the experimental data provides calibration curves that can be used to determine the water content with percentage relative error up to 70% for a water volume fraction of 0.06, but less than 10% for water volume fractions greater than 0.06. Furthermore, the methodology works over a wide range of volume fractions.

## 1. Introduction

In the oil industry, the determination of the water content in the crude oil extracted from the reservoir is important for the subsequent processes. From the physical properties relevant for controlling and improving the industrial process, the water volume fraction (ϕ) is very important because other properties strongly depend on it, such as density, viscosity, and stability. Usually, the limit on the amount of water allowed in crude oil for transportation and refining is less than 0.5% of the weight [1,2].

Several approaches have been used for the characterization of water–petroleum emulsions. Light [3] and neutron scattering [4], electron microscopy [5], and nuclear magnetic resonance [6] are important techniques, although restricted to dilute and non-opaque emulsions. Other techniques suitable for opaque media are based on X-rays [7] and gamma-rays [8]. The implementation of these techniques is complicated because they use ionizing radiations that are harmful to human health. Optical characterization techniques are of little use because the water-in-crude oil emulsions are opaque. An exception is LIBS (laser-induced breakdown spectroscopy) [9,10], which performs optical spectroscopy of a very small amount of material vaporized by a pulsed power laser. LIBS is a practically non-destructive technique, requires no sample preparation, and works with almost any material (solid of any hardness, liquids, even in aerosol form, etc.). However, the measurement is very localized and superficial, which can be a disadvantage in the case of emulsions. In addition, high-energy pulsed lasers can cause ocular damage. Electrical methods based on the measurement of impedance, permittivity and capacitance are probably the best established [11,12] in practical applications. However, these methods are strongly affected by the salinity and conductivity of the medium.

Ultrasound is an interesting technique because it is useful in opaque media, the acoustic waves are non-ionizing radiation, the technique is completely non-destructive and can be non-invasive, and the equipment required is relatively simple and cheap [13]. Despite all these advantages, practical applications in the industry require prior solutions for some problems. Due to the presence of the discrete phase of the emulsion, the propagation of ultrasonic waves is a complicated phenomenon. Additional modes other than longitudinal, such as thermal and shear modes, depending on the frequency, concentration, and droplet size, can become relevant [14]. As a consequence, high dispersion and attenuation may occur, such that the received signal can disappear. However, under certain conditions, ultrasound allows the emulsion to be characterized in detail. A well-known application is the determination by ultrasonic spectrometry of the concentration and droplet size distribution [15]. This technique is important for the study of contrast agents in contrast-enhanced ultrasound imaging and for other biotechnological applications. However, its use is restricted to dilute emulsions, and water-in-crude oil emulsions are, in general, concentrated [16].

For an emulsion with small droplet sizes compared to the wavelength, the wave propagation behavior is similar to that observed in a homogeneous medium. Under these conditions, ultrasonic wave propagation is efficient, but the information provided is limited. However, the ultrasound is still useful to indirectly estimate the water content in water-in-crude oil emulsions [17]. Ultrasonic parameters (such as propagation velocity and attenuation) have been used for emulsion characterization and monitoring [18,19]. Techniques based on the arrival time of the ultrasonic pulse are generally more stable and precise than those based on the amplitude [20,21].

The extraction of information from the acquired waveforms is essential for any characterization or monitoring application. Previous works use propagation velocity or amplitude-based parameters, such as cross-correlation and wave energy. The propagation velocity is a stable property when the droplet size is small compared to the wavelength. In these conditions, the measurement of the water content in the emulsion is feasible. This was demonstrated in a previous work [17], where a linear behavior of the propagation velocity as a function of the water volume fraction was observed. This result is interesting because the Urick mixture model predicts quadratic behavior. The determination of the water content by this method showed an error of less than 8.8%. In the case of amplitude-based parameters, the cross-correlation showed a strong and monotonic variation for ϕ < 0.2. However, the data show relatively high dispersion. The wave energy is a relatively stable parameter and it is easy to calculate, but it only provides a good resolution at low concentrations. The temperature effect in the range from 20 °C to 30 °C was studied in [20], showing that the propagation velocity provides perfectly distinguished curves at each temperature. Furthermore, the curves intersect at a volume fraction close to 0.57, a behavior predicted by the Urick mixture model. On the other hand, the cross-correlation and wave energy methods showed little variation with temperature. Another recent work used a delay-line-based measurement cell with a working frequency of 500 kHz to measure density and attenuation in water-in-crude oil emulsions [22]. The relatively low frequency used was intended to reduce the effect of the disperse phase on the propagation of ultrasonic waves. However, the parameters based on the amplitude of the signal, necessary to calculate the density and the attenuation, showed high dispersion.

As on-line and real-time applications require a stable parameter, insensitive to physical changes in the emulsion, the slope of the phase spectrum seems to meet this requirement. The use of algorithms based on the slope of the phase spectrum has been reported in the literature to accurately calculate the propagation velocity for material characterization [21,23,24,25,26]. The phase slope provides an accurate way to determine the time delay and thus the propagation velocity, which is useful in dispersive media where the ultrasonic pulse is distorted as it propagates [27]. The slope of the phase spectrum has also been used to determine the propagation velocity in plant leaves [28], which is a property directly related to water content. The determination of the water content in plant leaves is important in agriculture and ultrasound has gained relevance in this topic in the last years [29]. In a recent work from the medical area, the phase slope of ultrasonic shear waves was used for assessing the inflammation of the heart muscle [30].

This work proposes the use of the slope of the phase spectrum as a measurable parameter for determining the water content in water-in-crude oil emulsions. The results reported here demonstrate that there has been an improvement in the methodology of processing the signals obtained with the measurement devices developed in previous works [16,17,20,31]. Measurements for water volume fraction up to 0.48, including the pure water case, and three test temperatures (20 °C, 25 °C and 30 °C) were carried out using two different ultrasonic configurations: transmission–reception device (TRD) and backscattering device (BSD). In both cases, results showed the phase slope depends on the water content. Moreover, it is a stable property, almost insensitive to changes in the droplet size of the emulsion. The fitting of a simple model has the potential to be used as a calibration curve in practical applications.

## 2. Experimental Methods

### 2.1. Ultrasonic Configuration

**Figure 1:**
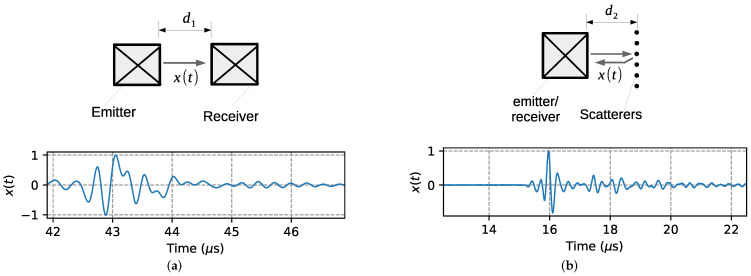
Experimental configurations and waveforms of (**a**) transmission–reception and (**b**) backscattering modes. (**a**) TRD. (**b**) BSD.

Figure 1 shows the experimental configuration of the ultrasonic tests in the cases of transmission–reception device and backscattering device, including the acquired waveform. The transmission–reception device (TRD) consists of two 4-MHz ultrasonic transducers with a square radiating area of 10 × 10 mm, facing each other and separated by a distance $d_{1}=30$ mm [20,22]. The backscattering device (BSD) consists of a row of rigid bars (scatterers) located in front of a commercial 3.5 MHz transducer with a circular radiating area with a 0.75-inch diameter. The 1.6 mm diameter bars are separated by 4 mm and the distance between the radiating face of the transducer and the row of bars is $d_{2}=11.5$ mm. The scatterers provide a sharp reflection of the waves that are received by the same transducer working in pulse-echo mode. These devices are simple, inexpensive, and can be inserted into any container or tube where the measurement is desired. These devices were developed in previous works for the monitoring of water-in-oil emulsions and further details can be found in [20,31].

**Figure 2:**
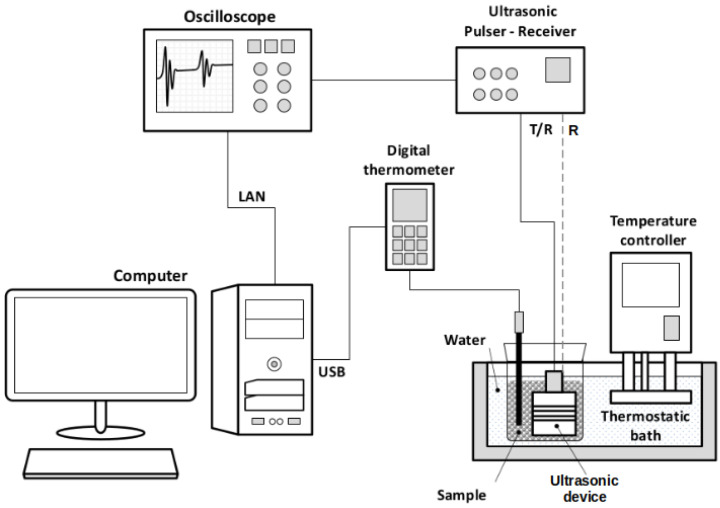
Experimental setup.

Figure 2 shows the experimental setup used for the acquisition of the ultrasonic signals. The measurements were made in a thermostatic bath with an accuracy of 0.1 °C (Huber CC-205B, Offenburg, BW, Germany). The transducer was driven in a transmission–reception device or pulse-echo modes using an ultrasonic pulser/receiver (Olympus Panametrics 5077-PR, Waltham, MA, USA). The digital oscilloscope (Agilent Technologies 5042, Santa Clara, CA, USA) was used to digitize the ultrasonic signals. The sample temperature was measured using a digital thermometer (DeltaOHM HD2107.2, Caselle di Selvazzano, PD, Italy). Both the oscilloscope and the digital thermometer were connected to a desktop computer, allowing the simultaneous acquisition and storage of the ultrasonic signals and temperatures.

The emulsion was stored in an 800 mL beaker partially immersed in the thermostatic bath. The samples were obtained from an initial volume of crude oil and then water was added and the sample was emulsified using a dispersing machine (IKA Labortechnick, model T25, Staufen, BW, Germany) at 8600 rpm, to obtain the new concentrations. After each emulsification step, the sample was left to rest for 5 min to remove most of the air bubbles and stabilize the temperature again. Next, the ultrasonic sensor and the thermometer were inserted into the beaker and data acquisition began. The acquisition time was 110 s and 15 min for the TRD and BSD, respectively. The test temperatures (*T*) were 20 °C, 25 °C, and 30 °C within a range of ±0.3 °C throughout the tests.

### 2.2. Signal Processing

The real-valued signal x(t) represents the ultrasonic waveform obtained in the acquisition process. The Fourier transform of the signal is:

(1) $$X\left(f\right)=F\left\{x\left(t\right)\right\}=\int_{-\infty }^{\infty }x\left(t\right)e^{-j2\pi ft}dt$$

where F is the Fourier operator, *t* is the time, *f* is the frequency, and *j* is an indeterminate satisfying $j^{2}=-1$ [32]. If the waveform is preserved, but there is a time delay (δ) in the arrival time, the time-shift property of the Fourier transform establishes that:

(2) $$F\left\{x\left(t-\delta \right)\right\}=e^{-2\pi jf\delta }F\left\{x\left(t\right)\right\}$$

The magnitude of the term $e^{-2\pi jf\delta }$ is always 1 and the magnitude spectrum does not change. On the other hand, the phase of the term $e^{-2\pi jf\delta }$ as a function of frequency is linear where the slope depends on δ [23].

The phase refers to the argument of the Fourier transform of x(t):

(3) ψ(f)=arg{X(f)}

and:

(4) $$m=\frac{d\psi }{df}$$

is the slope, which has values in the interval (−π,π) [23]. The relative phase slope is defined by:

(5) $$m_{r}=m-m_{ref}$$

where $m_{ref}$ is the phase slope measured in a reference case.

In this work, the reference case was pure oil because its physical properties vary depending on some factors, such as the origin reservoir and the extraction technique, among others. For a practical application of the method, the reference measurement can be obtained from an oil sample from the reservoir with all the water removed by one of the methods used for this purpose. However, the use of water as a reference fluid can be advantageous from an experimental point of view. The measurement with the reference sample must be performed at the temperature of interest, using the same time window and signal processing method. The $m_{ref}$ used in Equation (5) is the average value of the phase slopes obtained at test times (110 s for TRD and 15 min for BSD) of the pure oil for each of the test temperatures.

The signals acquired with TRD and BSD in water at 20 °C have −20 dB frequency bands of 0.5<f<4.7 MHz and 0.9<f<5.5 MHz, respectively. For the characterization of the emulsions, the frequency band between 1.0 and 5.0 MHz was chosen. The error in the measurement depends on the error in the determination of the phase slope. For non-dispersive materials, the wider the bandwidth, the smaller the error [28]. The signals were centered and the Fourier transforms were calculated using the Goertzel algorithm [33] with 200 points to represent the frequency range from 1 to 5 MHz and zero-padding to improve phase accuracy.

### 2.3. Data Fitting

The determination of the amount of water in the water-crude oil emulsion can be done by measuring the phase slope and using a calibration curve. For this purpose, it is proposed the fitting of the experimental data to the power function in a suitable frequency band:

(6) $$m_{r}=a\phi^{p},$$

where *a* and *p* are the fitting parameters and ϕ is the water volume fraction.

## 3. Results

**Figure 3:**
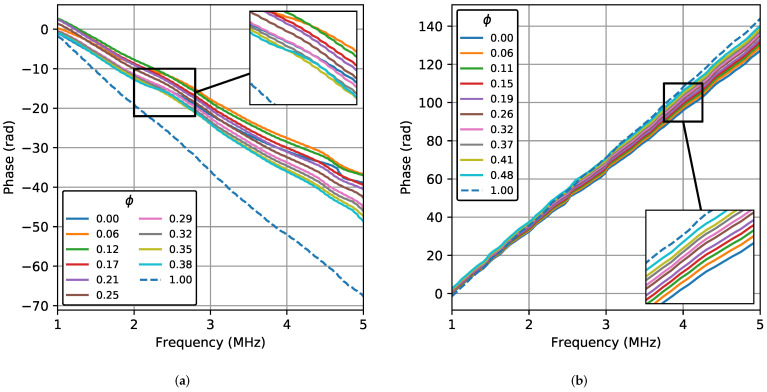
Phase of the Fourier transform as a function of frequency for all the water concentrations at 20 °C in the transmission–reception (TRD) and backscattering (BSD) cases. (**a**) TRD. (**b**) BSD.

Figure 3 shows the phase (ψ) as a function of the frequency of the signal obtained with the TRD and BSD in the 1–5 MHz frequency band for all the water concentrations. The presented results are the average of 30 acquisitions. In the TRD, the phase behavior is almost linear with decreasing values that lead to a negative slope. Although some curves overlap, a different slope is observed at each concentration. In the BSD, the linear behavior is more evident, with non-overlapping curves and increasing values leading to a positive slope. The sign of the slope can be negative or positive depending on the signal delay and the time window used. The delays due to the change in the propagation velocity are small, so the slope value varies within a narrow range. Figure 3 shows only the results for 20 °C because the behavior is identical for the other temperatures in both TRD and BSD. Just small differences in phase slopes were observed because the tests were performed at different temperatures.

**Figure 4:**
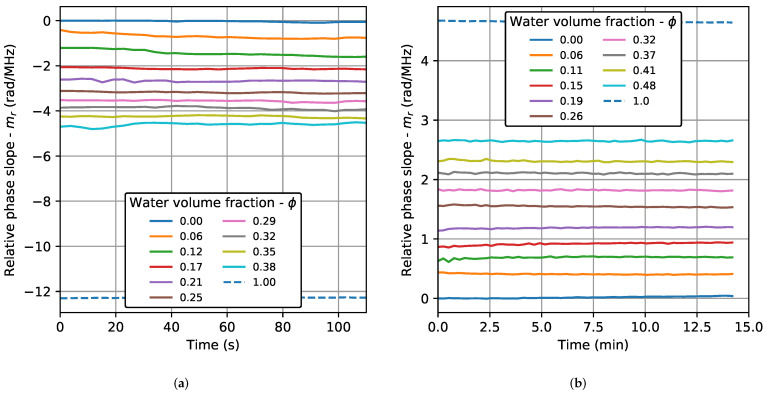
Relative phase slope as a function of the test time for all the concentrations at 30 °C: transmission–reception (TRD) and backscattering (BSD) cases. (**a**) TRD. (**b**) BSD.

Figure 4 shows the relative phase slope ($m_{r}$) as a function of the test time for all the concentrations at 30 °C in the TRD and BSD. In both cases, an almost constant behavior is observed, with values that increase depending on the amount of water in the emulsion. In the beginning, the variations observed are a consequence of the changes that occur in the first stage after the emulsification process, due to the heating and the air bubbles generated by the dispersing machine. So, these variations become smaller towards the end of the test time, when the emulsion becomes more stable. This condition was better observed in the TRD. The results show that the slope of the phase is a parameter that depends to a greater extent on the water content, being little affected by the droplet size of the dispersed phase. The observed behavior was the same for the three test temperatures. However, the variations at the beginning of the test were slightly higher for 30 °C. Only the results for 30 °C are shown. Results of parameters based on the amplitude of the signals (cross-correlation and wave energy) and on the propagation velocity as a function of time, reported in previous works [17,20], have a higher dispersion and time dependence, showing the advantage of the phase slope methodology.

**Figure 5:**
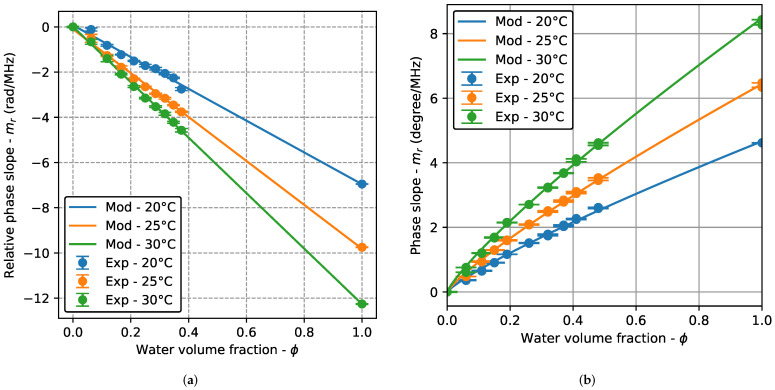
Relative phase slope as a function of the water volume fraction for the three test temperatures: transmission–reception (TRD) and backscattering (BSD) cases. Experimental results are the mean and standard deviation of 50 acquisitions and the solid lines are the fitting results. In the BSD case, two identical tests performed at each temperature are shown. (**a**) TRD. (**b**) BSD.

Figure 5 shows the relative phase slope ($m_{r}$) as a function of the water volume fraction (ϕ) for the three test temperatures (20 °C, 25 °C, and 50 °C). Two identical tests were performed at each temperature. As the values of $m_{r}$ are similar and the standard deviations small, the points are almost superimposed, making it look like a single measurement at the used plot scale. The experimental results are the mean and standard deviation of 30 acquisitions and the solid lines are the results obtained from the adjustment of Equation (6). In the TRD, a monotonic and almost linear variation of $m_{r}$ is observed, with perfectly differentiated curves at each temperature and a small standard deviation. The linear behavior is corroborated by the fitting results, as the value of the parameter *p* in (6) is very close to 1.0 for the three temperatures. The result for pure water (ϕ=1.0) agrees with the fitted line. In the BSD, the behavior is also monotonic (with small standard deviations) with distinct curves for each temperature. However, no linear behavior is observed and the value of the parameter *p* was 0.84 for the three temperatures. In this case, the pure water results are also in agreement with the measurements of the emulsions.

The monotonic behavior with a small standard deviation allows the adjustment of the simple model of Equation (6) in both TRD and BSD, providing calibration curves that can be used to determine the water content in industrial applications. Additionally, the position on the curve of the pure water results suggests that the method can be used over the full range of ϕ. This is an interesting feature because high volume fractions (oil-in-water emulsions) may appear in some processes. For example, a technique for transporting heavy crude oil consists of reducing the viscosity by generating a crude oil-in-water emulsion [34,35].

Calibration curves can be used to determine the water volume fraction from the phase slope. By comparing the results with the actual volume fraction of the emulsions, the percentage relative error (*e*) in the measurement can be calculated. For TRD, results show an error up to 70% for the lowest concentration (ϕ=0.0625). For ϕ>0.0625, the error decreases, with values up to 10%. For the BSD, e<27% for ϕ=0.0600, e<12% for ϕ=0.1100 and e<7% for ϕ>0.1100 and the results are similar for both tests. These results correspond to all the test temperatures (20 °C, 25 °C, and 30 °C). The error is higher for small volume fractions, as expected, and the same behavior is observed for the three test temperatures. The error values for all measurements were included in Table A2 of Appendix A.

The percentage of relative errors reported in a previous work [17], where the propagation velocity was used as the measurement parameter, was up to 46%. However, these high error values were observed in the entire range of measured volume fractions (ϕ<0.5), being a consequence of the higher dispersion of the propagation velocity results. Therefore, the methodology based on the phase slope provides better results than one based on the propagation velocity calculated in the time domain.

The deviations in the measurement are a consequence of errors in the determination of the phase slope. These errors come from experimental problems, such as low SNR due to high attenuation in the medium and instabilities in electronic equipment. In addition, the presence of the water droplets in the dispersed phase makes the medium dispersive to a certain extent, causing distortion in the waveform and, consequently, non-linear behavior of the phase spectrum.

The two ultrasonic devices used showed similar results. The main difference is better linearity and slightly higher resolution in the TRD. However, the BSD is cheaper, more robust, and easier to install in pipelines or tanks. Moreover, the signal processing is simpler and it could be performed by a microcontroller.

## 4. Conclusions

The slope of the phase spectrum is a viable tool for determining the water content in water-in-crude oil emulsions. The behavior was similar for both kinds of ultrasonic measurement cells used: a transmission–reception device (TRD) and a backscattering device (BSD). These cells were used to determine the delay time for water volumes up to 48%, as well as for pure water (100%), all in relation to the pure oil, at three test temperatures.

Results as a function of the test time showed the slope of the phase spectrum depends on the water content and it is a stable property, as long as the wavelength is greater than the droplets. On the other hand, results as a function of the water content show curves that vary monotonically, with a low standard deviation.

The data fitting using a simple power function provides calibration curves that allow the determination of the water volume fraction in practical applications. A linear behavior is observed when the transmission–reception device was used, but when the backscattering device was used, there was a non-linear behavior. The measurement was relative to the pure oil, which was used as a reference. The water could also be used as a reference.

Although the tests were performed for water volume fractions up to 0.48, pure water results are also consistent, with phase slope values located at the expected position in the calibration curves. This suggests that the methodology may be useful across the complete range of volume fractions, which makes it a complete option for industrial applications. Nonetheless, the need to obtain reference values, as well as the effects of temperature and droplet size, may limit the application of the technique in an industrial environment. Additional research on this topic is required.

The slope of the phase spectrum method, together with the expertise previously developed in the field of ultrasonic sensors, may make the measurement of water content in water-oil emulsions feasible in industrial environments. In order to test this possibility, future measurements will be made under conditions similar to those found in the industry, which surely implies a wider range of temperatures. The most likely approach is a system working on a bypass line. For this purpose, several systems need to be integrated, temperature control is required, and a droplet size smaller than the wavelength must be guaranteed.

## Appendix A. Non-Linear Regression and Measurement Error

Table A1 presents the values of the parameters *a* and *p* of Equation (6) obtained by non-linear regression. Parameter *a* has units of rad/MHz and parameter *p* is dimensionless. The results show different values for each temperature and repeatability can be observed in the BSD case. The parameter *p* has values very close to 1.0 and 0.84 for TRD and BSD, respectively. The phase slope can be positive or negative depending on the time delay of the received ultrasonic pulses. The fitting procedure was carried out using the scipy.optimize module of Python.

**Table A1:** Values of the model parameters *a* and *p* obtained by non-linear regression. In the BSD case, results are reported for two measurements.

Temperature	*a*	*p*
TRD		
20 °C	−6.9622	1.0227
25 °C	−9.7375	0.9814
30 °C	−12.2532	1.0001
BSD		
20 °C	0.0954	0.8445
	0.0993	0.8363
25 °C	0.1279	0.8527
	0.1387	0.8368
30 °C	0.1775	0.8395
	0.1770	0.8362

Table A2 presents the percentage relative error (*e*) obtained in the determination of the water volume fraction using the calibration curves. These curves were obtained using the parameters *a* and *p* shown in Table A1.

**Table A2:** Percentage relative errors (*e*) in the measurement of the water volume fraction (ϕ) in the emulsion using the calibration curve provided by the Equation (6) for both BSD and TRD and the three test temperatures. In the BSD case, results are reported for two measurements.

ϕ	20 °C		25 °C		30 °C	
TRD						
0.0625	70.4041		26.4452		13.8292	
0.1176	4.6524		1.9950		2.7766	
0.1667	10.2659		2.8685		2.3575	
0.2105	6.3868		5.7392		2.2519	
0.2500	1.5024		3.1947		2.7868	
0.2857	4.2620		1.1445		0.7446	
0.3182	4.5298		2.6452		1.3889	
0.3478	4.4829		2.0681		1.0110	
0.3750	7.4999		0.9280		0.4948	
1.0000	0.1632		0.0982		0.0247	
BSD						
0.0600	20.2329	19.1616	21.1338	26.6978	6.1881	27.4079
0.1100	12.3778	11.4234	6.6847	7.9421	10.3712	10.7466
0.1500	3.9190	6.5761	1.5081	2.8876	2.0349	2.2837
0.1900	1.8640	0.1621	2.4705	1.3688	2.0694	4.3459
0.2600	1.8753	0.5787	2.3939	0.5221	1.2479	0.3442
0.3200	2.7644	0.9325	0.8057	1.6980	0.5353	0.3600
0.3700	0.7847	2.0431	0.1885	2.0249	0.2879	1.5062
0.4100	2.7916	3.0380	0.5728	2.3719	3.3208	2.3886
0.4800	3.7018	4.1368	0.5338	2.1370	1.0392	0.8134
1.0000	1.0947	1.3451	0.2340	1.0841	0.6192	0.7981
